# Encapsulation of NEM Memory Switches for Monolithic-Three-Dimensional (M3D) CMOS–NEM Hybrid Circuits

**DOI:** 10.3390/mi9070317

**Published:** 2018-06-23

**Authors:** Hyun Chan Jo, Woo Young Choi

**Affiliations:** Department of Electronics Engineering, Sogang University, Seoul 04107, Korea; jhc10337@naver.com

**Keywords:** CMOS–NEMS, NEMS, NEM memory switch, encapsulation, M3D

## Abstract

Considering the isotropic release process of nanoelectromechanical systems (NEMSs), defining the active region of NEM memory switches is one of the most challenging process technologies for the implementation of monolithic-three-dimensional (M3D) CMOS–NEM hybrid circuits. In this paper, we propose a novel encapsulation method of NEM memory switches. It uses alumina (Al_2_O_3_) passivation layers which are fully compatible with the CMOS baseline process. The Al_2_O_3_ bottom passivation layer can protect intermetal dielectric (IMD) and metal interconnection layers from the vapor hydrogen fluoride (HF) etching process. Thus, the controllable formation of the cavity for the mechanical movement of NEM devices can be achieved without causing any damage to CMOS baseline circuits as well as metal interconnection lines. As a result, NEM memory switches can be located in any place and metal layer of an M3D CMOS–NEM hybrid chip, which makes circuit design easier and more volume efficient. The feasibility of our proposed method is verified based on experimental results.

## 1. Introduction

**Figure 1:**
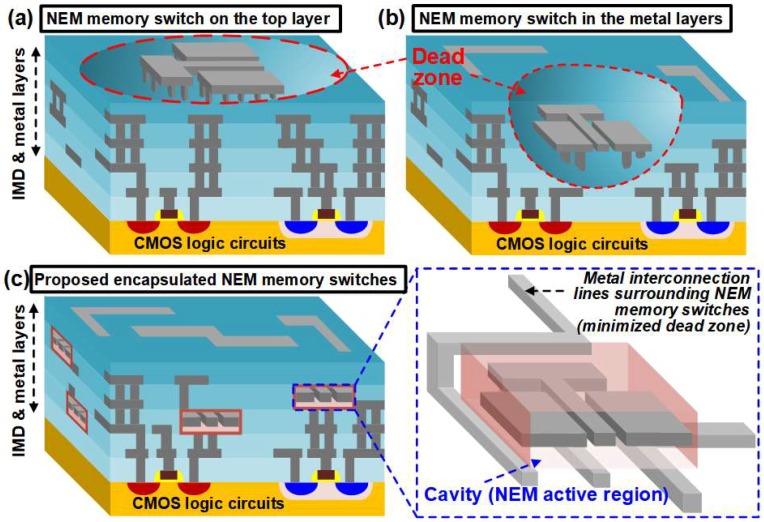
Conceptual views of (**a**) a nanoelectromechanical (NEM) memory switch only on the top layer, (**b**) a NEM memory switch in the CMOS back-end-of-line (BEOL) metal layers and (**c**) the proposed encapsulated NEM memory switches for monolithic-three-dimensional (M3D) CMOS–NEM hybrid circuits.

Complementary metal-oxide-semiconductor–nanoelectromechanical (CMOS–NEM) hybrid circuits have been researched intensively thanks to their unique advantages: low power consumption, high performance, low fabrication cost and high chip density [1,2,3,4,5,6,7,8,9]. Some pioneering experimental results of CMOS–NEM hybrid circuits have been reported [2,5]. They have NEM devices on the top of a chip or in CMOS back-end-of-line (BEOL) metal interconnection layers. For the implementation of monolithic-three-dimensional (M3D) CMOS–NEM hybrid circuits, the release process is important to form the atmospheric or vacuum environment for the mechanical motion of NEM memory switches whose operating mechanisms have already been explained elsewhere [1,2]. Generally, the release process is performed by using vapor hydrogen fluoride (HF) etching. By using the vapor HF etching, the inter-metal-dielectric (IMD) layers such as the tetraethyl orthosilicate (TEOS) layers, which surround NEM devices, can be effectively removed with high selectivity toward metal layers [10]. However, a conventional release process using vapor HF etching can cause catastrophic influences on IMD and metal interconnection layers because it is an isotropic etching process: NEM structures and adjacent metal interconnection lines collapse due to the widespread removal of IMD layers. Thus, as shown in Figure 1a,b, it is difficult to place the metal interconnection lines around NEM memory switches, which will be called the “dead zone” in this manuscript. The existence of the dead zone makes M3D CMOS–NEM hybrid circuit design difficult and volume inefficient.

To minimize the dead zone surrounding NEM devices, this manuscript proposes a novel CMOS-process-compatible encapsulation method as shown in Figure 1c. In the proposed method, NEM memory switches are encapsulated by alumina (Al_2_O_3_) bottom/top passivation layers. The TEOS lower/upper sacrificial layers encapsulated by the Al_2_O_3_ bottom/top passivation layers are selectively removed by vapor HF etching while the rest of the regions are protected. Thus, the controllable formation of a cavity is feasible for the mechanical movement of NEM devices without damaging CMOS baseline circuits and metal interconnect lines. From now, this cavity will be called the “active region” of a NEM memory switch. To sum up, because our proposed encapsulation method defines the active regions of NEM devices without generating dead zones, they can be placed in any metal interconnection layers. To confirm the proposed method, prototype encapsulated NEM memory switches are implemented. 

## 2. Encapsulation Process

**Figure 2:**
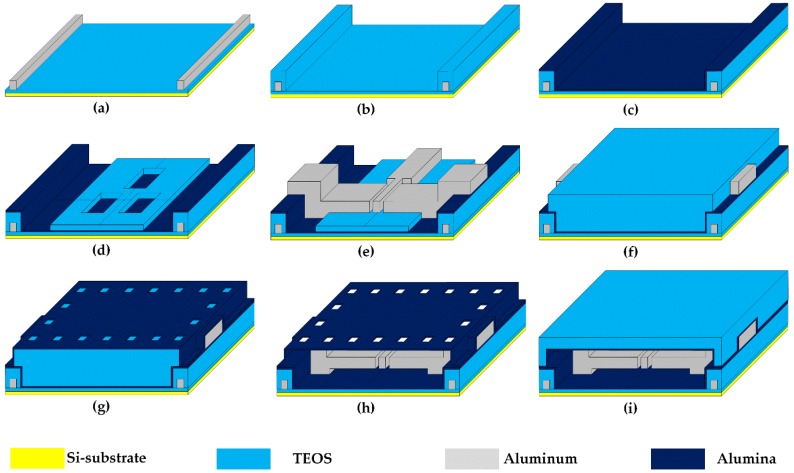
Key process steps of the encapsulated nanoelectromechanical (NEM) memory switches. (**a**) Al deposition and patterning for the formation of metal interconnection lines; (**b**) Tetraethyl orthosilicate (TEOS) deposition and patterning for inter-metal-dielectric (IMD) formation; (**c**) Al_2_O_3_ bottom passivation layer deposition; (**d**) Lower TEOS sacrificial layer deposition and patterning; (**e**) Al deposition and patterning for the formation of a NEM memory switch; (**f**) Upper TEOS sacrificial layer deposition and pattern; (**g**) Al_2_O_3_ top passivation layer deposition and etch hole formation; (**h**) Removal of the lower/upper sacrificial layers through etch holes by using vapor hydrogen fluoride (HF) etching; (**i**) TEOS deposition for cavity sealing.

Figure 2 shows the key process steps of the encapsulated nanoelectromechanical (NEM) memory switches. First, a 50-nm-thick silicon dioxide (SiO_2_) layer is grown by wet oxidation. Then, a 500-nm-thick aluminum (Al) layer is sputtered and patterned by inductively coupled plasma (ICP) etching. The Al patterns correspond to the metal interconnect lines of CMOS baseline circuits. Third, a 500-nm-thick tetraethyl orthosilicate (TEOS) inter-metal-dielectric (IMD) layer is deposited and patterned by plasma-enhanced chemical vapor deposition (PECVD) and magnetically enhanced reactive ion etching (MERIE) processes, respectively, to define the active regions of NEM memory switches. Subsequently, a 200-nm-thick Al_2_O_3_ bottom passivation layer is deposited by a multisputtering process. The Al_2_O_3_ bottom passivation layer protects the metal interconnection lines and IMD layers from the following vapor hydrogen fluoride (HF) etch at atmospheric pressure [11,12,13]. Fifth, a 200-nm-thick TEOS layer is deposited as a lower sacrificial layer. Next, a 500-nm-thick Al layer is deposited and patterned to form NEM memory switches. During the patterning process, the 85-nm-wide airgap between the movable cantilever beam and selection lines is formed by a focus ion beam (FIB) process while the rest of the patterns are defined by a conventional stepper. Seventh, a 500-nm-thick TEOS layer is deposited and patterned as an upper sacrificial layer. It should be noted that the active regions of NEM memory switches are defined and filled by the lower and upper sacrificial layers. Eighth, a 200-nm-thick Al_2_O_3_ top passivation layer is deposited to encapsulate the active regions of NEM memory switches. Subsequently, small-sized etch holes are patterned on the Al_2_O_3_ top passivation layer by the FIB process. Tenth, the lower and upper TEOS sacrificial layers are removed through the etch holes by vapor HF etching at 40 ˚C and 15 min. Finally, a thick TEOS IMD layer is deposited on the Al_2_O_3_ top passivation layer to form the cavity surrounding NEM memory switches which acts as the active region. The encapsulated active regions are in the vacuum condition depending on TEOS deposition conditions. This encapsulation method is fully CMOS-process-compatible, which can be easily applied to the fabrication of M3D CMOS–NEM hybrid circuits.

**Figure 3:**
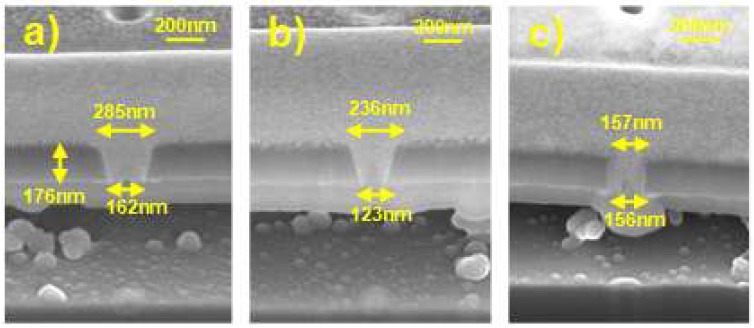
Cross-sectional scanning electron microscopy (SEM) images of etch holes with the variation of the beam current and target diameter of the focus ion beam (FIB) process. (**a**) Aspect ratio = 0.79 when beam current is 50 pA and target diameter is 160 nm; (**b**) Aspect ratio = 1.01 when beam current is 10 pA and target diameter is 160 nm. (**c**) Aspect ratio = 1.14 when beam current is 10 pA and target diameter is 80 nm.

For cavity formation, the etch holes should have the aspect ratio high enough to prevent TEOS from filling the cavity again through the etch holes. Figure 3 shows scanning electron microscopy (SEM, Thermo Fisher Scientific, Waltham, MA, USA) cross-sectional images of etch holes. In order to form the etch holes with various aspect ratios, two FIB process conditions have been adjusted: beam current and target diameter. The aspect ratio of the etch holes in Figure 3a–b are measured to be 0.79 (beam current = 50 pA and target diameter = 160 nm) and 1.01 (beam current = 10 pA and target diameter = 160 nm), respectively. It is interesting that two different layers are observed below the Al_2_O_3_ top passivation layer in those two cases. The former is a thin TEOS layer which is originated from the unwanted TEOS inflow through the etch holes. It is problematic in that it prevents the motion of a cantilever beam of a NEM memory switch. On the contrary, the latter results from the redeposition process during the FIB sample cutting process for SEM measurement, which does not exist in the main samples [14]. Thus, to suppress TEOS inflow, the aspect ratio of the etch holes needs to be increased. If the aspect ratio is increased up to 1.14 (beam current = 10 pA and target diameter = 80 nm) as shown in Figure 3c, no unwanted TEOS inflow is observed. Only the redeposition layer originated from the FIB sample cutting process is formed under the Al_2_O_3_ top passivation layer. 

## 3. Results and Discussion

**Figure 4:**
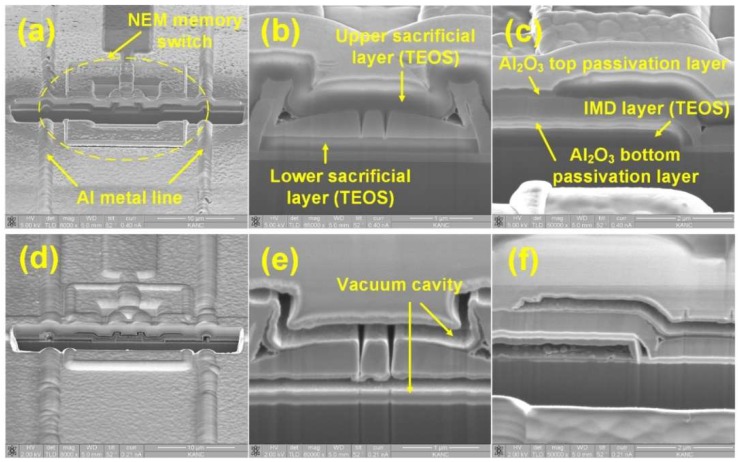
(**a**) Nanoelectromechanical (NEM) memory switch and metal interconnection lines; (**b**) NEM memory switch and (**c**) metal interconnection lines before vapor hydrogen fluoride (HF) etching; (**d**) NEM memory switch and metal interconnection lines; (**e**) NEM memory switch and (**f**) metal interconnection lines after vapor HF etching.

**Figure 5:**
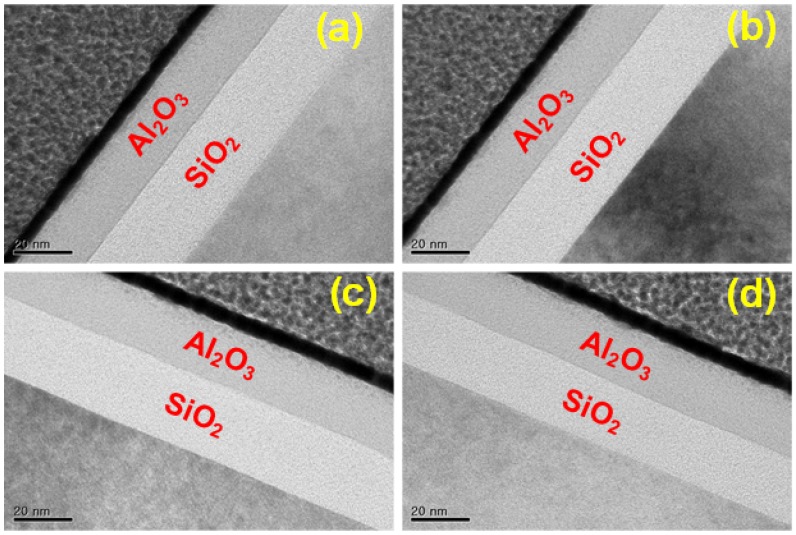
Transmission electron microscopy (TEM) images of an atomic layer deposition (ALD)-deposited Al_2_O_3_ layer (**a**) before and after (**b**) 1-min, (**c**) 5-min and (**d**) 15-min vapor hydrogen fluoride (HF) etching.

Figure 4 shows the SEM images of the fabricated NEM memory switch encapsulated in a cavity. Figure 4a–f show the NEM memory switches before and after vapor hydrogen fluoride (HF) etching, respectively. The active region of the encapsulated NEM memory switch is formed well next to the metal interconnection lines, as shown in Figure 4. Figure 4b,c confirm that Al_2_O_3_ top and bottom passivation layers wrap the NEM memory switch and lower/upper tetraethyl orthosilicate (TEOS) sacrificial layers. Figure 4d–f show that the TEOS lower/upper sacrificial layers are successfully removed by vapor HF etching. In Figure 4e, it is confirmed that the sacrificial layers are completely removed by vapor HF without damaging the cavity regions. This forms the active region of the NEM memory switch, allowing activation between metal layers. Especially, Figure 4e shows the successful implementation of the NEM memory switch in a cavity. On the other hand, Figure 4f shows that the inter-metal-dielectric (IMD) layer out of the cavity is also removed by vapor HF etching, which means that the Al_2_O_3_ bottom passivation layer fails to protect the IMD layer from vapor HF etching. It is because vapor HF can penetrate into the Al_2_O_3_ layer following grain boundaries if the Al_2_O_3_ layer is formed by the sputtering process. Thus, in order to increase the film density of the Al_2_O_3_ passivation layer, an atomic layer deposition (ALD) process is used rather than a sputtering process. Figure 5a–d show the transmission electron microscopy (TEM) images of the test sample using a 20-nm-thick ALD-deposited Al_2_O_3_ layer before and after 1-, 5- and 15-min vapor HF etching at 40 ˚C, respectively. As predicted, it is observed that the SiO_2_ IMD layer is completely protected by the ALD-deposited Al_2_O_3_ layer. 

**Figure 6:**
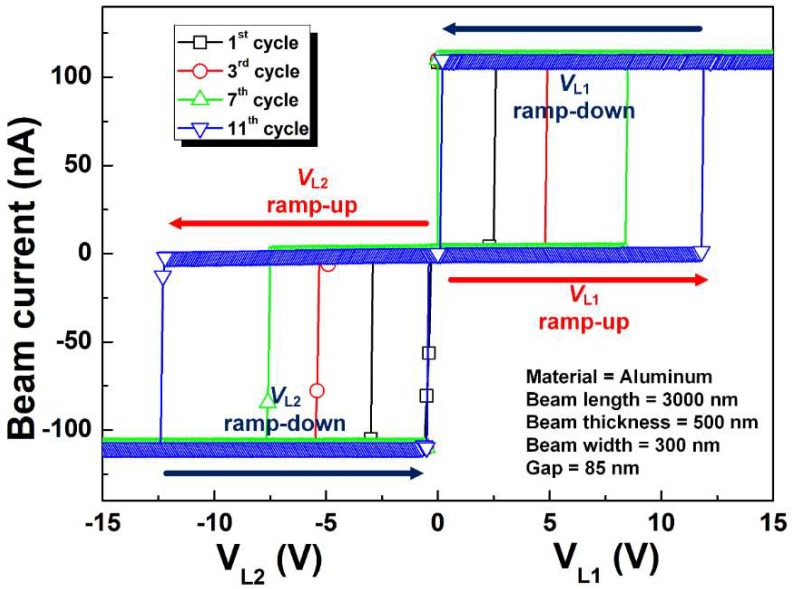
Current vs voltage curves of the fabricated nanoelectromechanical (NEM) memory switch encapsulated in a cavity.

Figure 6 shows the current vs voltage curves of the fabricated NEM memory switch encapsulated in a cavity. It shows the reasonable nonvolatile switching operation between selection line 1 (*L*_1_) and selection line 2 (*L*_2_). The endurance cycle number is ~11 times due to the weak mechanical property of aluminum. In the first switching operation, the voltage difference between the movable cantilever beam and *L*_1_ (*V*_*L*1_) becomes higher than the pull-in voltage (*V_pull-in_*), and then the movable cantilever beam is stuck onto *L*_1_, which is called State 1. In this case, because the adhesion force (*F_ad_*) is larger than the restoring spring force of the movable cantilever beam (*F_r_*), the movable beam remains in contact with *L*_1_ even when *V*_*L*1_ is 0 *V* [15]. Thus, the nonvolatile data signal storage can be achieved. In the second switching operation, the voltage difference between the movable cantilever beam and *L*_2_ (*V*_*L*2_) becomes higher than the switching voltage (*V_swit_*), and then the location of the beam tip is changed from *L*_1_ to *L*_2_, which is called State 2. During the measurement, maximum current level was limited to suppress microwelding effects. Poor endurance cycle number can be improved by downscaling the dimension of NEM memory switches and changing beam materials [15,16].

## 4. Conclusions

In this work, a fabrication method to encapsulate an NEM memory switch for CMOS–NEM hybrid circuits is proposed by using a commercial CMOS process and materials. Specification of the stable encapsulated NEM memory switch is successfully confirmed based on the prototype fabrication and measurement results. By applying the proposed method confirmed in this work, the active regions of NEM memory switches can be formed without damaging CMOS baseline circuits as well as the metal interconnect lines. Because NEM memory switches can be located in any place and metal layer, the design of M3D CMOS–NEM hybrid chips can be easier and more volume efficient. It should be noted that our proposed encapsulation method can be applied to any kind of NEM device, including NEM switches, as long as they are fabricated by a CMOS backend process. For more uniform and reliable processes, a reduced-pressure vapor HF etcher can be used rather than the atmospheric-pressure vapor HF etcher used in this work. Therefore, the proposed fabrication process can lay the groundwork for commercialization of M3D CMOS–NEM hybrid circuits.
